# Papaverine adjuvant therapy for microcirculatory disturbance in severe ulcerative colitis complicated with CMV infection: a case report

**DOI:** 10.1007/s12328-019-00974-y

**Published:** 2019-04-03

**Authors:** Yu Tian, Yue Zheng, Jinpei Dong, Jixin Zhang, Huahong Wang

**Affiliations:** 1 Gastroenterology Department of Peking, University First Hospital, Beijing, China; 2 Pathology Department of Peking, University First Hospital, Beijing, China

**Keywords:** Ulcerative colitis, Papaverine, Microcirculatory disturbance, Confocal laser endomicroscopy

## Abstract

Ulcerative colitis has hypercoagulable state and high risk of thrombosis; so mucosal disturbance of microcirculation may be mediate and amplify the inflammation of ulcerative colitis. A 56-year-old female patient was admitted in hospital for discontinuously mucous bloody stool for more than 1 year. Ulcerative colitis was determined after colonoscopy and pathologic examination. Mesalazine was effective during the year, but her symptoms recurred three times due to her bad compliance. One month before admission, the patient had severe recurrence after mesalazine withdrawal. At this time, the result of quantitative fluorescence PCR of colonic histic CMV-DNA was 1.6 × 10^4^ copies/mL positive, CMV colitis was accompanied. After 4 weeks of ganciclovir and 6 weeks of mesalazine usage and nutrition support, the symptoms of diarrhea and abdominal cramp did not improve; stool frequency was more than twenty times a day. Probe-based confocal laser endomicroscopy revealed local microcirculation disturbance. Papaverine 90-mg slow drip for at least 10 h a day was added. The symptoms dramatically disappeared after 3 days of papaverine treatment. The patient had yellow mushy stool 2–3 times a day. Pathological findings showed diffuse submucosal hemorrhage and transparent thrombosis in capillaries. Treatment of microcirculatory disturbance in severe UC is a promising adjuvant therapy. Confocal laser endomicroscopy may be an effective method for microcirculation judgment.

## Background

Ulcerative colitis (UC) has hypercoagulable state, the risk of thrombosis increases. It suggests that the formation of microthrombosis may be one of the important pathogeneses of UC [1]. Especially in patients with severe active UC, submucosal thrombosis is often found by pathological examination, and capillary microthrombosis can also be seen where inflammation is not obvious [2]. There is evidence that coagulation activation may in turn mediate and amplify inflammatory cascades in inflammatory bowel disease (IBD) [3]. Treatment of UC with low-molecular-weight heparin (LMWH), which has anticoagulant effect, can improve clinical symptoms [4]. Similar findings were observed in animal models of colitis with vasodilator papaverine (PAP) [5]. It is important to determine the changes of mucosal blood flow associated with chronic inflammation.

Dextran sulfate sodium (DSS)-induced colitis was one of the classic UC animal models. Some research had found that DSS administration elicited capillary vessel disruption before epithelial cells damage appeared. So, mucosal microcirculatory disturbance was recognized as the triggers for DSS-induced colitis [6]. As well as in animal models of colitis, extensive angiogenesis and microcirculation reorganization occurred in the inflamed area in IBD patients [7]. The ischemic condition promoted additional inflammatory cell recruitment and sustained the inflammatory response [8].

In this case, on the basis of adequate treatment for UC, the occurrence of mucosal microcirculatory disturbance was found by confocal laser endomicroscopy (CLE) and papaverine (PAP) therapy achieved remarkable effect.

## Case presentation

A 56-year-old female patient was admitted in hospital for discontinuously mucous bloody stool for more than 1 year and aggravation with fever for 1 month. More than 1 year ago (April 2017), mucous loose and bloody stool appeared in the patient three times a day without fever, fatigue, rash or joint pain. UC was diagnosed by colonoscopy and pathological examination. Then, mesalazine was given 2 g daily for 2 weeks and the symptoms improved quickly. One month later, the symptoms of the patient were completely relieved, and mesalazine was discontinued.

Five months before admission symptoms recurred (February 2018), UC E3 was diagnosed again by colonoscopy. Symptoms completely relieved after the therapy for mesalazine 3 g plus 0.5 g of mesalazine suppository daily for about 1 month.

One month before admission, when the patient went out for travel and stopped using mesalazine, the mucous blood stool gradually increased to more than 20 times a day, dark red blood stool when symptom got serious, accompanied by lower abdominal pain before defecation, general weakness, loss of appetite and 10-kg weight loss within 1 month. During these days, there were no other special medication usage and no rashes appeared. Laboratory examination showed normal liver and kidney function, but CRP and ESR were greatly elevated with 93.23 mg/L and 50 mm/h, and Hb and Alb obviously reduced to 99 g/L and 26.1 g/L. Abdominal CT showed thickening of the whole colon wall, especially in the left side. Fever occurred 2 days before admission with a maximum of 38 °C. The patient has no special hobby and family history. Hysteromyomectomy was performed 11 years ago, and at that time CT revealed calcification of the left kidney.

**Table 1:** Laboratory data on admission

Blood cells		Blood chemistry		Serological test		Pathogen	
WBCs	5.9 × 10^9^/L	TP	60.2 g/L	IgG	8.84 g/L	Blood bacteria culture	Negative
Eo	0.2 × 10^9^/L	Alb	31.3 g/L	IgA	2.65 g/L	Stool C. Diff A/B	Negative
RBCs	4.05 × 10^12^/L	T-bil	12.4 μmol/L	IgM	0.57 g/L	PCT	0.09 ng/mL
Hb	104 g/L	AST	10U/L	ANA	Negative	Stool smear for TB	Negative
Platelets	336 × 10^9^/L	ALT	6U/L	ANCA	Negative	Stool smear for fungus	Negative
PT	13.5 s	LDH	153U/L	SF	3.5 μmol/L	Stool bacteria culture	Negative
APTT	31.6 s	ALP	50U/L	TIBC	18.5 μmol/L	T-spot TB	Negative
Fibrinogen	3.84 g/L	γ-GT	15U/L			Blood CMV-DNA	Negative(< 500 copies/mL)
D-Dimer	0.42 mg/L	TCHO	1.84 mmol/L			Blood EBV-DNA	Negative(< 500 copies/mL)
ESR	42 mm/h	Glu	5.33 mmol/L			G/GM test	Negative

*WBCs* white blood cells, *Eo* eosinophile granulocyte, *RBCs* red blood cells, *Hb* hemoglobin, *PT* prothrombin time, *APTT* activated partial thromboplastin time, *ESR* erythrocyte sedimentation rate, *TP* total protein, *Alb* albumin, *T-bil* total bilirubin, *AST* aspartate aminotransferase, *ALT* alanine aminotransferase, *LDH* lactate dehydrogenase, *ALP* alkaline phosphatase, *γ-GT* γ-glutamyltransferase, *TCHO* total cholesterol, *Glu* glucose, *Ig* immunoglobulin, *ANA* antinuclear antibody, *ANCA* anti-neutrophil cytoplasmic antibodies, *SF* serum ferritin, *TIBC* total iron binding capacity, *C. diff* Clostridium difficile, *PCT* Procalcitonin, *T-spot* TB tuberculosis interferon-γ release assay, *CMV* cytomegalovirus, *EBV* Epstein–Barr virus

On admission, *T* 37.2 °C, P 92 beat per minute, superficial lymph nodes were not enlarged. Cardiopulmonary examination was normal. Abdominal palpation with no tenderness points and bowel sounds were 4 times per minute. Abdominal ultrasonography showed thickening of the whole colon wall and mild splenomegaly. Admission diagnosis: UC severe activity E3.

In the first week of admission, no positive results were found in fecal smears for fungi and TB bacteria, and clostridium difficile toxin A/B was also negative. PPD was negative. Blood culture showed no bacterial growth. Quantitative fluorescence PCR of blood CMV-DNA and EBV-DNA were less than 500 copies/mL. G and GM tests were negative. PT was normal but D-dimer was positive (laboratory data are shown in Table 1). Nutritional support therapy (mainly enteral nutrition) and mesalazine 4 g/day oral and 0.5 g for rectal administration every night were added, accompanied by meropenem plus tinidazole for anti-infection empirical therapy, but symptoms did not improve.

**Fig. 1:**
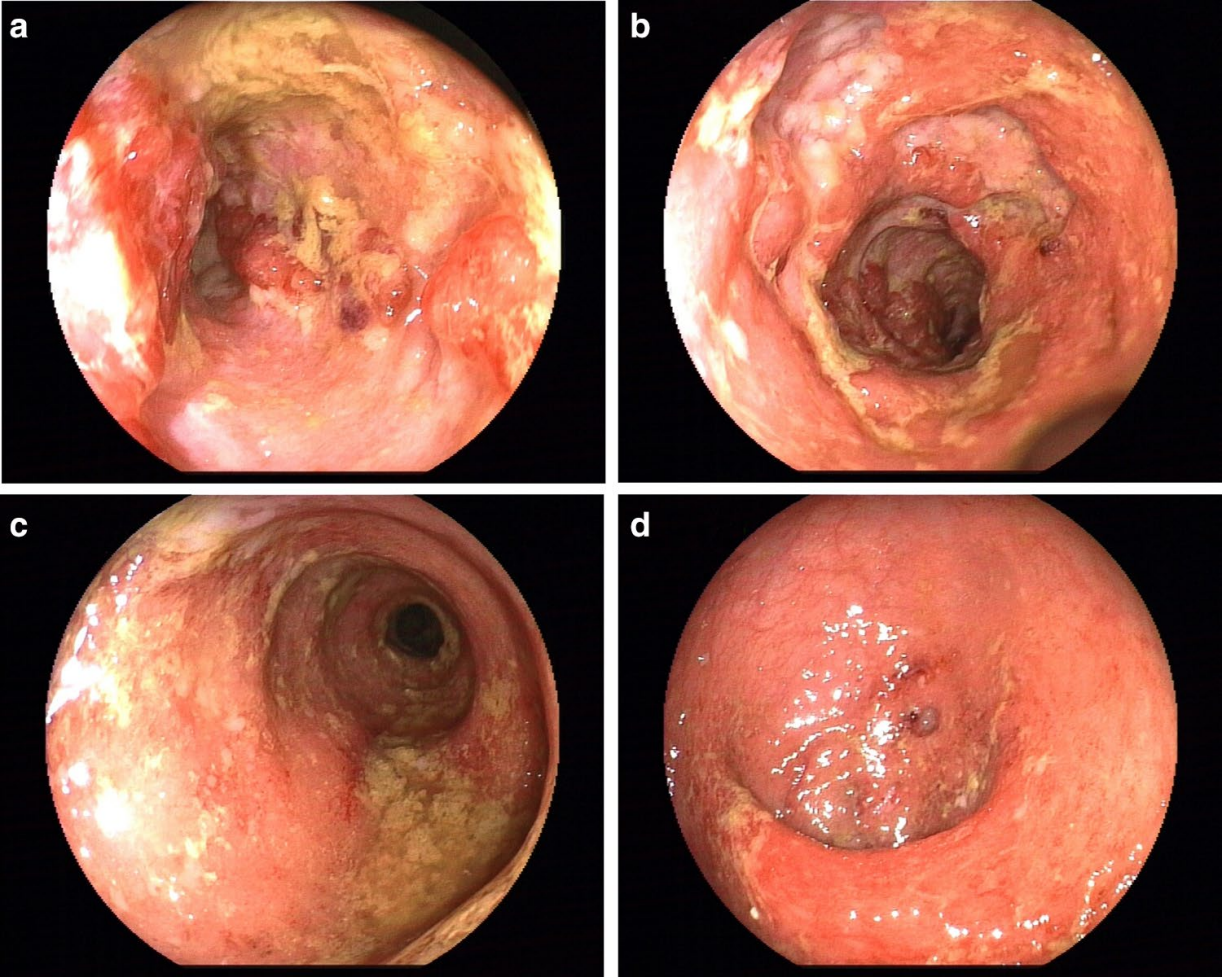
**a** Transverse colon, deep and large longitudinal ulcers. **b** Transverse colon, deep and large ulcers. Biopsy with CMV-DNA in mucosal tissue. **c** Sigmoid colon, diffused edema, congestion, and ulcers of the mucosa. **d** Rectum, diffused edema, congestion, and ulcers of the mucosa

At the second week of admission, treatment had not changed, but extensive edema, erosion and ulcers of the colonic mucosa were observed by colonoscopy (see Fig. 1a–d). Multipoint pathological biopsy with CMV/EBV-DNA in mucosal tissue was performed.

At the third week of admission, colonic pathological examination determined UC diagnosis, but at the same time, quantitative fluorescence PCR of colonic histic CMV-DNA 1.6 × 10^4^ copies/mL positive was detected. According to the clinical characters, the diagnosis of severe UC complicated with CMV colitis was made. Intravenous full-dose ganciclovir was added and antibiotics were discontinued.

At the 6th week of admission (after 3 weeks of antiviral therapy), the general symptoms improved obviously, but diarrhea was still serious, more than 20 times a day with yellow loose stool, sometimes blood stool and lower abdominal cramp before defecation. During the period, the coagulation function was normal and mesalazine was continued.

**Fig. 2:**
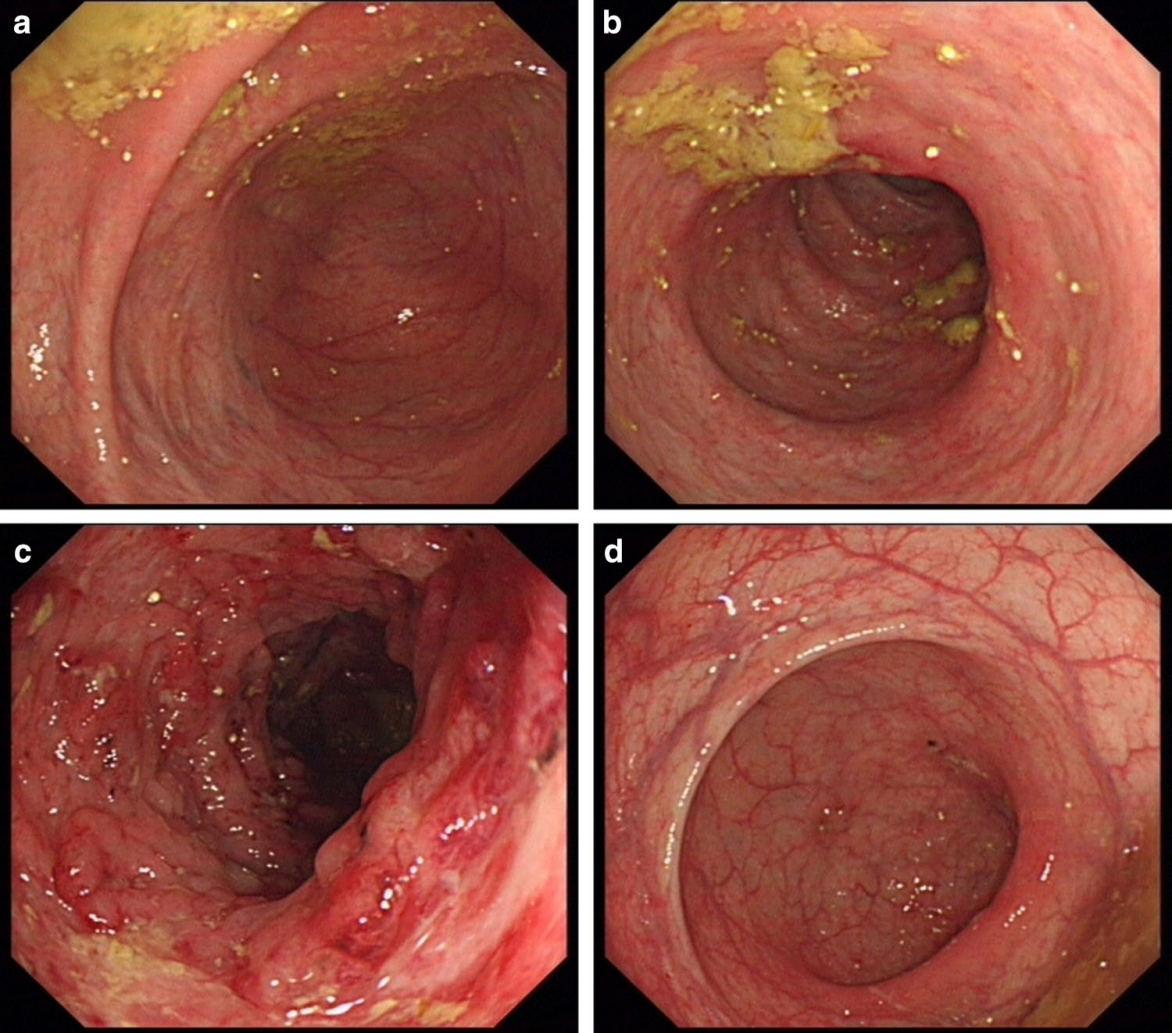
**a** Cecum, mucosal ulcer scars have seen. **b** Ascending colon, mucosal edema with confused vascular network. **c** Transverse colon, mucosal edema, congestion, showing nodular shape. pCLE images were obtained here. **d** Rectum, mucosa almost returned to normal

**Fig. 3:**
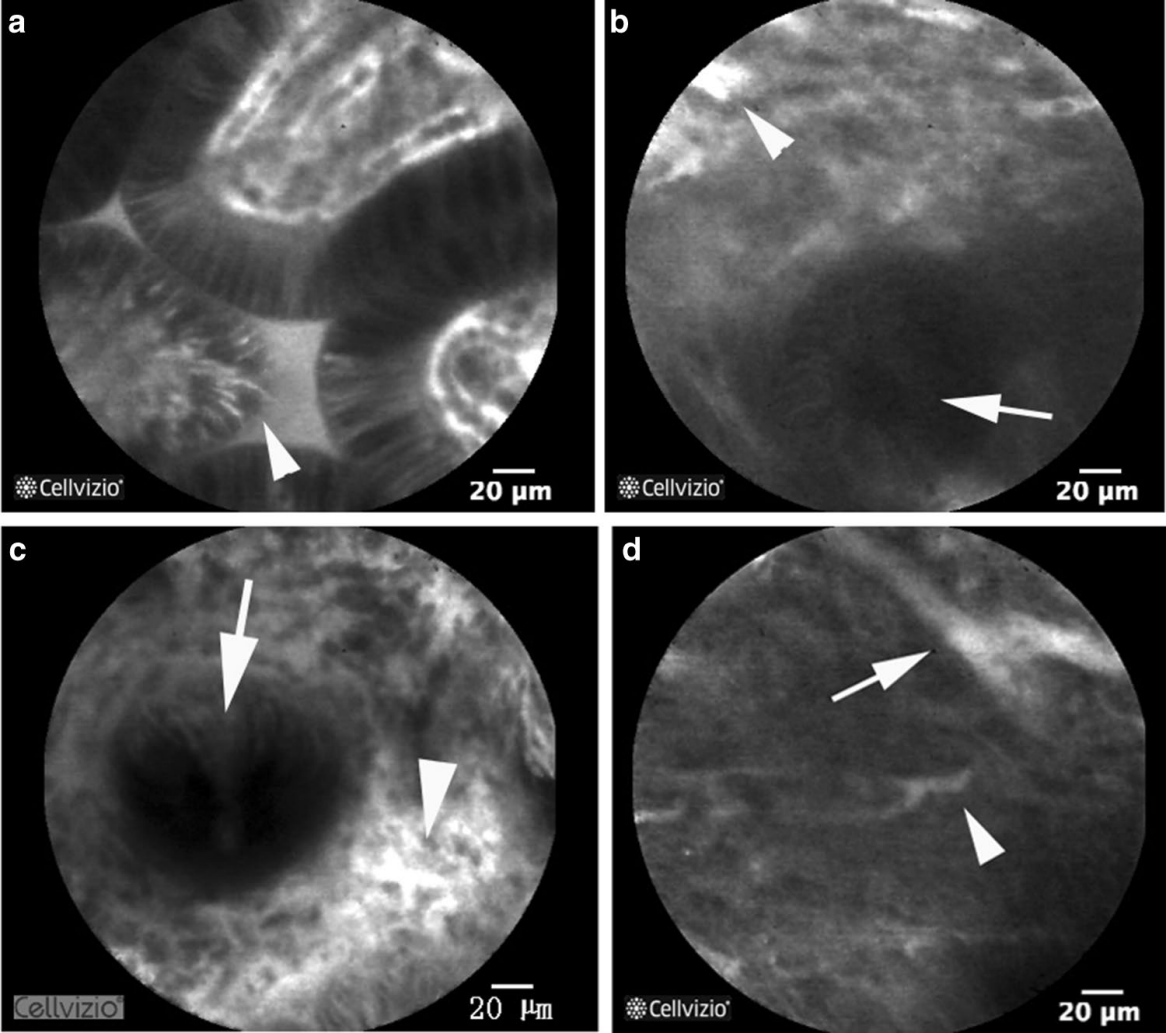
**a** pCLE image of the terminal ileum, increased epithelial gaps and fluorescein leakage (indicated by arrowhead). **b** pCLE image of the transverse colon, fluorescein leakage in the perivascular area (indicated by arrowhead), but not in the intact glandular cavity (indicated by arrow). **c** pCLE image of the transverse colon, fluorescein leakage in the perivascular area (indicated by arrowhead), but not in the intact glandular cavity (indicated by arrow). **d** pCLE image of the transverse colon, vascular diameter was uneven (indicated by arrow and arrowhead) and blood flow interruption phenomenon was always seen

**Fig. 4:**
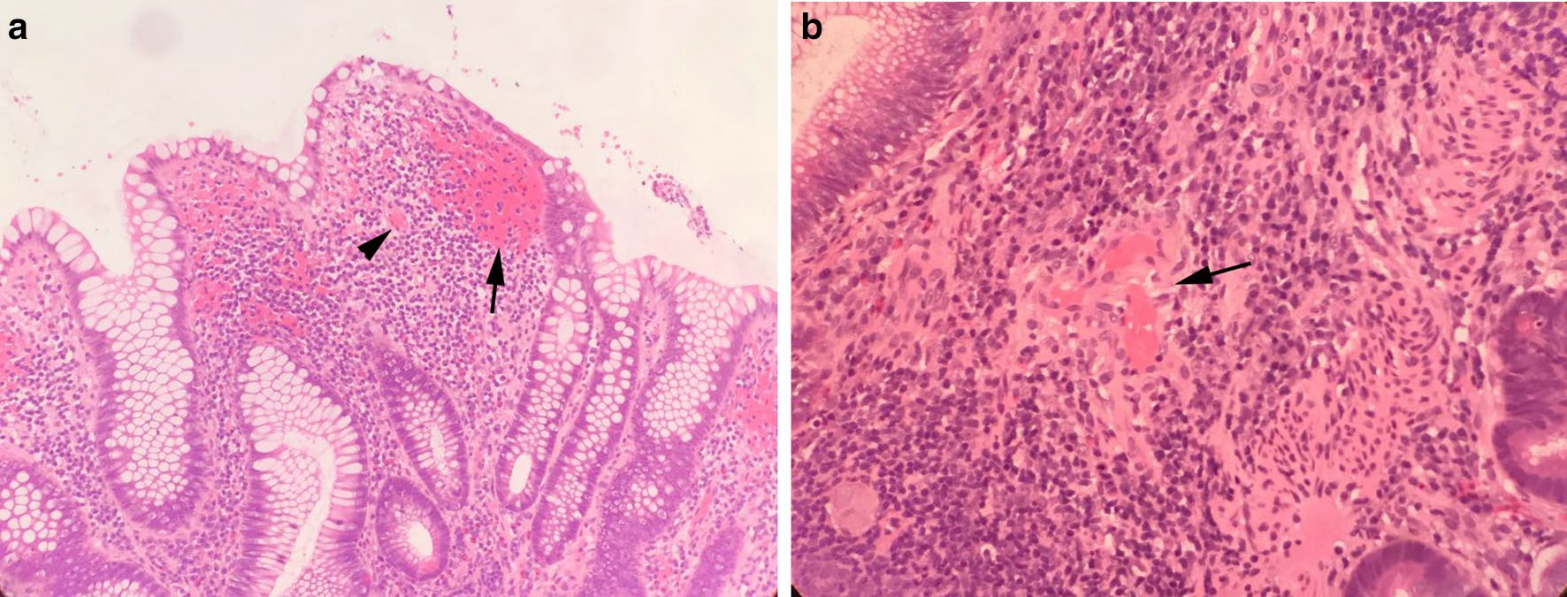
**a** Pathological image of transverse colon HE stain, submucosal hemorrhage (indicated by arrow) and transparent thrombus in capillaries (indicated by arrowhead) in superficial mucosa. **b** Pathological image of transverse colon HE stain, inflammatory cell infiltration and transparent capillary thrombosis in deep mucosa (indicated by arrow)

At the 7th week of adminssion, ganciclovir (after 4 weeks of antiviral therapy) was stopped, but the symptom of diarrhea did not improve. Colonoscopy was preformed again which revealed that inflammation was obvious in the transverse to splenic flexure colon (see Fig. 2a–d). Probe-based confocal laser microendoscopy (pCLE) revealed local microcirculation disturbance (see Fig. 3a–d). PAP 90-mg slow drip for at least 10 h a day was added. The symptoms of abdominal pain and diarrhea dramatically disappeared after 3 days of PAP treatment. The patient had yellow mushy stool 2–3 times a day. Pathological findings were consistent with pCLE images, with diffuse submucosal hemorrhage and transparent thrombosis in capillaries (see Fig. 4a, b).

At the nineth week of admission, PAP was stopped after a 10-day therapy. The patient’s symptoms were almost all relieved. She was discharged from hospital and continued to take mesalazine 4 g/day to treat the disease.

## Discussion

The diagnosis of the patient was clear for UC with CMV enteritis, which was in a period of severe activity. The main causes of the deterioration of the disease included the discontinuation of mesalazine, and CMV infection due to decreased immunity of exhaustion. Drug allergy can be excluded because of no special medication usage, rash and eosinophilia in the course of the disease. During the course of treatment, diarrhea symptoms remained prominent after 4 weeks of antiviral and 6 weeks of mesalazine treatment. At that time, colonoscopy found that the inflammation of colonic mucosa was generally improved, while the mucosal inflammation from transverse colon to splenic flexure was prominent. This site exactly known as watershed area is the most commonly affected site of colonic ischemia [9].

Confocal laser endomicroscopy has been used to determine the degree of inflammation in UC [10]. The advantage of CLE is that it can reflect colonic crypts, epithelial gaps, epithelial leakiness to fluorescein and the microcirculation of mucosal blood flow in vivo [11]. The average detect depth of probe-based CLE is 55–65 μm [12], just the level of deep intestinal mucosa capillary network can be observed [13]. For this patient, adjusting the degree of the probe close to the mucosa, especially to the edge of ulcer, hyperemia and erythema area, the changes of colonic mucosal blood flow can be clearly observed. Analyzing CLE images, the following characteristics are obtained: (1) epithelial gaps increased and fluorescein leakage at the end of ileum indicates the increase of intestinal mucosal permeability and decrease of intestinal barrier function. This image feature has been recognized in many studies [14]. (2) The capillary network in the interstitium around the gland of transverse colon mucosa is blurred, and the leakage of perivascular fluorescein is obvious, which indicates that the inflammation is obvious there. It is widely accepted that such a fluorescein leak suggests the apparently inflammation [15]. (3) There are a large number of intact glands in transverse colonic mucosal where inflammation is obvious but no fluorescein leakage in the intact glandular cavity. This phenomenon suggests that either the barrier function of these glands is normal, or the blood supply of these glands is relatively insufficient for fluorescein to leak out. It is unlikely that glands with good barrier function will appear in the prominent part of inflammatory response. So, it is reasonable to speculate that ischemia is happened in that glandular epithelium. And mucosal damage would be occurred if there were persistent hypoxias. These findings of pCLE were confirmed by pathology.

Local microcirculation disturbance can occur in acute or chronic stages of IBD [16, 17]. Animal model of IBD has shown that microcirculation disturbance occurs before inflammation relapses [6]. However, CLE images of the blood flow are easier to observe in chronic convalescent stage, because in acute stage, mucosal and vascular damage is prominent, massive necrotic tissue and obviously leakage of fluorescein affected the image acquisition. The microcirculation disturbance reflected in this case is the same as the periphery ischemic area after capillary injury in the literature [18]. Our team has observed some similar cases for microvascular changes in UC patients under CLE, and those changes were seen commonly. In this case, according to the absence of leukocytosis, splenomegaly, steroid refractory and colonic histic positive, UC combined with CMV infection was determined [19]. There might be a relationship between CMV infection and microcirculatory disturbance, because studies have found that CMV causes cell lysis of endothelial cells [20], and the incidence of CMV-infected thrombosis increased in clinical [21] [22]. Some cases of PE caused by UC combined with CMV infection have been reported [23]. All these suggest that microcirculation disturbance is more prominent in UC patients combined with CMV infection.

The importance of vascular involvement in UC has been known for many years [24]. Intestinal microcirculation has multiple crucial roles in the pathogenesis of UC, especially in angiogenesis [25]. Some studies [26] [27] demonstrated the presence of enhanced microvessel density in intestinal tissue of UC patients, which correlated with the disease activity. The functions of the microvessel need more attention. Symptom of the patient that almost twenty bowel movements a day accompanied with lower abdominal cramp was really a difficult problem before PAP treatment. On the first day after using PAP, the symptom of diarrhea eased rapidly. By the third day of using PAP, the daily frequency of defecation had decreased to its normal level, 2–3 times a day. The significant improvement in symptoms was related to the use of PAP, when other treatments were not adjusted.

Papaverine an opioid analogs can relieve the spasm of vascular smooth muscle [28]. It is mainly used to treat ischemia caused by spasm of blood vessels of the heart, brain and peripheral. PAP was more often used in gastroenterologist’s prescription to treat ischemic colitis. Its role has not been fully elucidated. At present, it is believed that it mainly inhibits the activity of cyclic nucleotide phosphodiesterase [29]. Some studies have found the anti-inflammatory effect of PAP by inhibiting ROS, leukocyte infiltration and inflammatory cytokines such as IL-1, IL-6 and TNF [30, 31]. Recently, some scholars have approved that PAP can not only inhibit transcription/production of proinflammatory factors but also promote the neuroprotective process by the study of lipopolysaccharide (LPS)-induced microglial activity, and these effects were mediated by NF-κB signaling pathway [32]. It is concluded that PAP might be a valuable anti-neuroinflammatory candidate. Although PAP has many anti-inflammatory and protective mechanisms, its rapid relief of abdominal pain and diarrhea symptoms in such a short time for this patient showed that relaxation of smooth muscle and improving blood supply were the most important factor, and of course, further researches are needed.

As anticoagulant therapy, LMWH has a definite effect on UC [33]. Studies have found that LMWH is targeted at intestinal microthrombosis [34]. Although there is no consensus on the use of PAP in the treatment of ischemic colitis [35], as a vasodilator, PAP becomes a routine therapy in our center because of its rapid relief of clinical symptoms, and its protective effect shown in DSS-induced colitis model [5]. The outstanding effect of improving intestinal microcirculation in this UC case shows an important mechanism of UC occurrence and development. It is reasonable to believe that improving the treatment of mucosal microcirculation may become a meaningful direction for IBD treatment and research in the future.

## Conclusion

Mucosal microcirculatory disorders in UC should be paid more attention, and improvement of microcirculation method at right time may become an important adjuvant therapy. pCLE may be an effective method for real-time observation of mucosal blood flow in vivo, which needs further study.

## Abbreviations

UC: Ulcerative colitis
DSS: Dextran sulfate sodium
IBD: Inflammatory bowel disease
CLE: Confocal laser endomicroscopy
CMV: Cytomegalovirus
EBV: Epstein–Barr virus
PAP: Papaverine
ESR: Erythrocyte sedimentation rate
CRP: C reactive protein
Hb: Hemoglobin
Alb: Albumin
CT: Computed tomography
TB: Tuberculosis
IL: Interleukin
TNF: Tumor necrosis factor
LPS: Lipopolysaccharide
NF-κB: Nuclear factor-κB
LMWH: Low-molecular-weight heparin
